# Burden Imposed by Heterologous Protein Production in Two Major Industrial Yeast Cell Factories: Identifying Sources and Mitigation Strategies

**DOI:** 10.3389/ffunb.2022.827704

**Published:** 2022-02-01

**Authors:** Louise La Barbera Kastberg, Ryan Ard, Michael Krogh Jensen, Christopher T. Workman

**Affiliations:** ^1^Department of Biotechnology and Biomedicine, Technical University of Denmark, Lyngby, Denmark; ^2^Department of Biology, University of British Columbia, Kelowna, BC, Canada; ^3^Novo Nordisk Foundation Center for Biosustainability, Technical University of Denmark, Lyngby, Denmark

**Keywords:** burden, yeast, heterologous protein production, strain engineering, metabolism, biotechnology

## Abstract

Production of heterologous proteins, especially biopharmaceuticals and industrial enzymes, in living cell factories consumes cellular resources. Such resources are reallocated from normal cellular processes toward production of the heterologous protein that is often of no benefit to the host cell. This competition for resources is a burden to host cells, has a negative impact on cell fitness, and may consequently trigger stress responses. Importantly, this often causes a reduction in final protein titers. Engineering strategies to generate more burden resilient production strains offer sustainable opportunities to increase production and profitability for this growing billion-dollar global industry. We review recently reported impacts of burden derived from resource competition in two commonly used protein-producing yeast cell factories: *Saccharomyces cerevisiae* and *Komagataella phaffii* (syn. *Pichia pastoris*). We dissect possible sources of burden in these organisms, from aspects related to genetic engineering to protein translation and export of soluble protein. We also summarize advances as well as challenges for cell factory design to mitigate burden and increase overall heterologous protein production from metabolic engineering, systems biology, and synthetic biology perspectives. Lastly, future profiling and engineering strategies are highlighted that may lead to constructing robust burden-resistant cell factories. This includes incorporation of systems-level data into mathematical models for rational design and engineering dynamical regulation circuits in production strains.

## Introduction

Biomanufacturing of heterologous proteins from genetically engineered cell factories is a growing industry. The first such biopharmaceutical product was approved by the FDA four decades ago (U.S. Food Drug Administration, 1982; Nielsen, 2013), and now over 300 different biopharmaceuticals are available (Walsh, 2018). Beyond delivering many life-saving drugs, biopharmaceutical protein production serves as an economically profitable industry representing an expanding multi-billion-dollar global market (Walsh, 2018; Mordor Intelligence, 2021). Apart from this, commercial protein products for industrial and research applications are also produced in engineered cells and likewise constitute a growing industry (Baghban et al., 2019). However, despite many previous and ongoing successes, continued bioengineering of cell factories is required for more robust and reliable production of heterologous protein.

Today, an assorted palette of living organisms with distinct advantages and disadvantages are used for industrial-scale heterologous protein production, including bacteria, fungi, and mammalian cell lines (Dumont et al., 2016). Depending on the protein product of interest, developers choose the most appropriate host organism based on quality and quantity (Porro et al., 2011). Yeast represents an attractive host for the production of many types of heterologous proteins when compared to other host systems.

The first heterologous protein product released to the market was human insulin produced in an engineered strain of *Escherichia coli* using recombinant DNA technology and branded as Humulin™ (U.S. Food Drug Administration, 1982). Since then, bacterial expression systems have been a popular choice for producing multiple heterologous proteins since they replicate quickly in cheap growth media and, in general, produce high protein titers (Karbalaei et al., 2020). On the other hand, they lack the capacity to perform necessary post-translational modifications required, especially for many pharmaceutical proteins to be active (Ghaderi et al., 2012). Most bacteria, including *E. coli*, are also unable to successfully secrete such proteins, requiring added cell harvesting, cell disruption, and product isolation steps before protein purification (Lalor et al., 2019). Thus, protein recovery from lysed bacteria cells often requires time-consuming and costly downstream processing (Vieira Gomes et al., 2018). Alternatively, mammalian cell lines can be used to achieve proper protein modifications, folding, and secretion for simpler downstream purification (Dumont et al., 2016). Although several valuable pharmaceutical proteins are expressed from mammalian cell platforms, including therapeutic monoclonal antibodies (Kunert and Reinhart, 2016; Walsh, 2018), these cells grow slower than microbial cell factories and are much more expensive to cultivate (Karbalaei et al., 2020).

Since yeast are unicellular eukaryotes, these fungi require less expensive culturing conditions and possess many of the post-translational and secretion pathways present in higher eukaryotes. As such, yeast cell factories are commonly used to produce diverse heterologous proteins for pharmaceutical, industrial, and research applications (Table 1, Baghban et al., 2019; Kulagina et al., 2021). The two most favored yeast production hosts are *Saccharomyces cerevisiae* and *Komagataella phaffii* (syn. *Pichia pastoris*) (Tripathi and Shrivastava, 2019) and will be the focus of this review.

**Table 1:** Examples of heterologous proteins produced in *Saccharomyces cerevisiae* (*Sc*) and *Komagataella phaffii* (*Kp*).

**Heterologous protein**	**Original host**	**Production host**	**Description**	**Reference**
**Pharmaceuticals**				
Insulin precursor	Human, porcine	*Sc, Kp*	Hormone used for treatment of diabetes	Zhu et al., 2009; Kazemi Seresht et al., 2013; Vanz et al., 2014; Chen et al., 2017; Wright et al., 2020; Shen et al., 2021
Immunoglobulin G (IgG)	Human	*Sc*	Antibody for monoclonal antibody therapy	de Ruijter et al., 2018
Aprotinin	Bovine	*Sc*	Antifibrinolytic protein that reduces blood loss and need for blood transfusion	Krogh et al., 2008
Growth hormone	Human	*Kp*	Peptide hormone for growth stimulation	Matthews et al., 2018
Granulocyte colony-stimulating factor	Human	*Kp*	Hormone-like protein for treatment of HIV-associated neutrophil defects	Zhang et al., 2006
**Industrial enzymes**				
Cel7A	*Talaromyces emersonii* (Fungus)	*Sc*	Cellobiohydrolase hydrolyzing cellulose from reducing end	Lamour et al., 2019
AppA	*Escherichia coli* (Bacterium)	*Kp*	Phytase used in animal feed	Navone et al., 2021
β-glucosidase	*Saccharomycopsis fibuligera* (Yeast)	*Sc*	Hydrolyzes cellobiose attacking non-reducing end	Van Rensburg et al., 2012
Cellobiohydrolase II	*Trichoderma reesei* (Fungus)	*Kp*	Cellobiohydrolase hydrolyzing cellulose from non-reducing end	Mellitzer et al., 2014
a-amylase	*Saccharomyces kluyveri* (Yeast)	*Sc*	Hydrolyzes alpha bonds in polysaccharides	Tyo et al., 2012; Huang et al., 2017, 2018
β−1,4-Xylanase II	*Trichoderma reesei* (Fungus)	*Sc*	Hydrolyzes polysaccharides into xylose	Görgens et al., 2001
Xylanase A	*Bacillus halodurans* (Bacterium)	*Kp*	Catalyzes hydrolysis of polysaccharides into xylose	
Lipase	*Bacillus thermocatenulatus* (Bacterium), *Rhizopus oryzae* (Fungus)	*Kp*	Hydrolysis and transesterification of triacylglycerols	Jordà et al., 2012; Cámara et al., 2016; Barrero et al., 2021
β-galactosidase	n/a	*Kp*	Catalyzes hydrolysis of cell wall pectin	Nie et al., 2014; Liu et al., 2016
Glucose oxidase	n/a	*Kp*	Glucose oxidation	Yu et al., 2020
Lignin peroxidase	*Phanerochaete chrysosporium* (Fungus)	*Kp*	Lignin oxidation	Majeke et al., 2020
β-aminopeptidase	*Sphingosinicella xenopeptidilytica* (Bacterium)	*Kp*	N-terminal nucleophile hydrolase	Heyland et al., 2011b

Despite many commercial success stories, challenges still remain when engineering heterologous protein production in yeast. Yeast cells have evolved to readily adapt to fluctuating environmental conditions, and may down-regulate protein production to match intracellular demands imposed by various external and internal conditions and stresses (Gasch, 2003). For production strains, this coordination is important to ensure a competitive trade-off between fitness and cost-intensive protein production which consumes cellular resources. Thus, resource intensive protein production can easily disrupt this delicate intracellular balance due to the redirection of cellular resources that are normally distributed among native cellular activities, including biomass formation and growth (Heyland et al., 2011b). Such production burden (Glick, 1995; Heyland et al., 2011b; de Ruijter et al., 2018), typically results in reduced protein titers, limiting process efficiency and production profitability.

Burden is not the only limitation that may negatively impact production of protein in yeast cells. Product toxicity, caused by protein aggregation for example, can also have an impact on the health and productivity of yeast cell factories (Eguchi et al., 2018; Chen et al., 2020; Romero-Suarez et al., 2021). However, not all heterologous proteins have toxic effects on host cells. In contrast, burden represents a universal challenge for heterologous protein production since it incurs resource demands for the host cells at potentially all levels of gene expression.

In this review, we will focus on burden conferred by heterologous soluble protein production in yeast cell factories from recombinant DNA replication through to the final secreted product. Since achieving desirable heterologous protein production is paramount, we will highlight recent strategies to mitigate burden in two of the most common yeast systems used for this purpose and propose new paths for future research. Although many concepts discussed here will be relevant to burden in other production organisms, the unique challenges presented by non-yeast systems remain outside the scope of this review.

### Two Favored Budding Yeast Production Strains

#### Saccharomyces cerevisiae

Several yeasts are used as host organisms for protein production, including *S. cerevisiae, Kluyveromyces lactis, Yarrowia lipolytica, Hansenula polymorpha, Ogataea polymorpha*, and *K. phaffii* (Rebello et al., 2018; Vieira Gomes et al., 2018; Kulagina et al., 2021). The budding yeast *S. cerevisiae* is among the most well-established organisms for heterologous protein production. Often called a “conventional yeast,” *S. cerevisiae* is highly domesticated and has been used by humans for thousands of years to produce bread and alcoholic beverages (Mattanovich et al., 2014; Patra et al., 2021). The extensive use of *S. cerevisiae* has facilitated its establishment as an important model organism for life sciences (Chen et al., 2020). Notably, it was the first eukaryotic organism to have its genome sequenced (Goffeau et al., 1996). Subsequent years of research have resulted in a well-annotated genome and an extensive genetic toolbox for *S. cerevisiae* production strain engineering.

Beyond genetic engineering, *S. cerevisiae* possesses many additional advantages for heterologous protein production. It possesses eukaryotic pathways to post-translationally process and secrete proteins to the extracellular medium, while simultaneously secreting few endogenous proteins in low amounts. *S. cerevisiae* can tolerate harsh growth conditions, such as low pH, and finally, it has gained the generally recognized as safe (GRAS) status allowing for easier approval processes (U.S. Food Drug Administration, 2018). Indeed, For these reasons, *S. cerevisiae* has become an established host for production of heterologous proteins such as human insulin, glucagon, and lignocellulosic enzymes (Table 1, Baghban et al., 2019; Kulagina et al., 2021).

#### *Komagataella phaffii* (syn. *Pichia pastoris*)

Other non-conventional yeasts have been explored for their suitability in heterologous protein production. An attractive alternative budding yeast is *K. phaffii*. Ecallantide, a drug used to treat hereditary angioedema, was the first pharmaceutic protein produced and approved in *K. phaffii* in 2009. It has also become established for industrial-scale protein production and manufacturing of insulin, human serum albumin, antibody fragments, phytases, and trypsin (Research Corporation Technologies, 2009, 2019). *K. phaffii* possesses many of the same characteristic advantages mentioned for *S. cerevisiae*, such as GRAS status (Vogl et al., 2013). Compared to *S. cerevisiae*, it can grow to higher cell densities and has a greater secretory capacity (Duman-Özdamar and Binay, 2021). Another important difference involves lower glucose uptake in *K. phaffii* compared to *S. cerevisiae* (Peña et al., 2018). *K. phaffii* is a crab tree-negative yeast, which can be an advantage over the crab tree-positive yeasts, e.g., *S. cerevisiae*, that can produce toxic levels of ethanol during aerobic cultivations (Cereghino and Cregg, 2000).

Another attractive feature of *K. phaffii* is its methylotrophic nature. It thrives on simple carbon sources like methanol, which is otherwise toxic to many other microorganisms including *S. cerevisiae* (Riley et al., 2016). Genetic engineering often exploits this unique feature by placing genes for desired heterologous proteins under the control of a strong methanol-inducible promoter from the *Alcohol oxidase 1* gene (*AOX1*). This allows for a biomass formation phase prior to an induced expression production phase, where the medium carbon source is switched from glycerol, glucose, or sorbitol to methanol (Celik et al., 2009; Noseda et al., 2013). Challenges such as inducer-toxicity, fire hazard, and heat production are related to the use of methanol, present concerns for large-scale production and have caused researchers to explore the engineering of methanol-free expression systems in *K. phaffii* (Wang et al., 2017). Despite its strengths, *K. phaffii* is less established and genetically characterized, meaning that metabolic and genome engineering of *K. phaffii* lags behind that of *S. cerevisiae* (Kalender and Çalik, 2020; Duman-Özdamar and Binay, 2021). However, genome sequences and annotations are available for the two most relevant *K. phaffii* strains GS115 and CBS7435 (De Schutter et al., 2009; Küberl et al., 2011; Love et al., 2016; Valli et al., 2016) and substantial progress in genetic engineering has in recent years enhanced heterologous protein production (Yang and Zhang, 2018). For example, employing the constitutive glyceraldehyde-3-phosphate dehydrogenase (GAP) promoter (Qin et al., 2011). Overall, continued advances help overcome important initial drawbacks of using *K. phaffii* as a production host and underpin the relevance of using it for industrial-scale heterologous protein production.

#### Common Challenges

Both of these yeast cell factories face similar challenges. For example, the challenge of N-glycan hyper-mannosylation of heterologous protein in *S. cerevisiae*, although less pronounced in *K. phaffii* (Mizukami et al., 2018; Vieira Gomes et al., 2018), remains problematic since it can render certain pharmaceutical proteins inactive, such as monoclonal antibodies (Jung and Kim, 2018). Yet, there are still opportunities to use yeast cell factories to produce some glycoprotein products. This requires either additional biochemical processing steps before they can be used as pharmaceutical proteins, or further glycoengineering of production strains with more humanized glycosylation patterns (Liu et al., 2018; Kulagina et al., 2021).

Together, these two budding yeasts represent highly attractive and cost-effective platforms for producing a plethora of different therapeutic and industrial proteins and ongoing research continues to enhance their performance. Despite their advantages, burden conferred by heterologous protein production remains a challenge encountered in all production hosts, and yeasts are no exception (Heyland et al., 2011a,b; Niklas et al., 2013; de Ruijter et al., 2018; Zou et al., 2018). Like the challenge of N-glycan hyper-mannosylation, we expect genome engineering will be critical for building more burden resilient production strains.

## Burden—The Competition for Cellular Resources

Heterologous protein production in yeast consumes a variety of cellular resources for expression, processing, and transport. This includes consumption of metabolic precursors, redox co-factors, and energy sources (Mattanovich et al., 2014; Klein et al., 2015; Zahrl et al., 2019). Such metabolic remodeling impacts cell growth and limits normal cellular metabolic processes, often curbing heterologous protein production (Glick, 1995; Kazemi Seresht et al., 2013). This reallocation was first defined as metabolic burden in bacteria (Glick, 1995) but the nomenclature describing the phenomenon in yeast varies from metabolic burden to protein burden or fitness burden (Harrison et al., 2012; Kafri et al., 2016; Deparis et al., 2017; Huang et al., 2017; Saeki et al., 2020; Garrigós-Martínez et al., 2021; Wright et al., 2021). In this review, we will refer to this concept simply as “burden.”

To what extent burden affects the cell is case-dependent, since heterologous proteins of differing size and biochemical complexity demand different resources from the host cell (Heyland et al., 2011b; de Ruijter et al., 2018). Therefore, the competition for resources varies depending on several factors, such as amino acid composition, post-translational modifications, metabolism of the host, and carbon availability (Heyland et al., 2011b; Tyo et al., 2012). Differing expression levels for any given heterologous protein can also have a difficult-to-predict impact on production. For example, Mellitzer et al. described different classes of heterologous proteins based on how gene dosage affected expression yield. They found that increasing gene copy number for some heterologous proteins resulted in higher active protein yields, while for others yields remained constant or even decreased (Mellitzer et al., 2012, 2014). Revealing the underlying mechanisms responsible for these differences would provide critical information about how burden is established in different production strains. In line with this, different engineering strategies will likely be needed to mitigate burden depending on the protein of interest (Gu et al., 2015). But first we must be able to assess the ways in which cells are burdened by heterologous protein expression.

Assessing burden imposed by heterologous protein production is crucial for engineering more resilient and higher-producing strains. But how is burden measured? Widely used approaches involve analyzing physiological parameters like cell growth rate, biomass yield, and respiratory capacity (Kazemi Seresht et al., 2013; Liu et al., 2014; de Ruijter et al., 2018). Apart from monitoring cells through these parameters, additional methods and -omics technologies have been applied in recent years to assess burden and its sources (Heyland et al., 2011b; Jordà et al., 2012; Liu et al., 2016; Huang et al., 2017; Wright et al., 2020). These methods provide measurements of the internal cellular state, including, but not limited to, metabolites, transcriptional regulation, translational efficiency, and carbon flux. Together, these approaches help describe the observed burdened phenotype.

**Figure 1:**
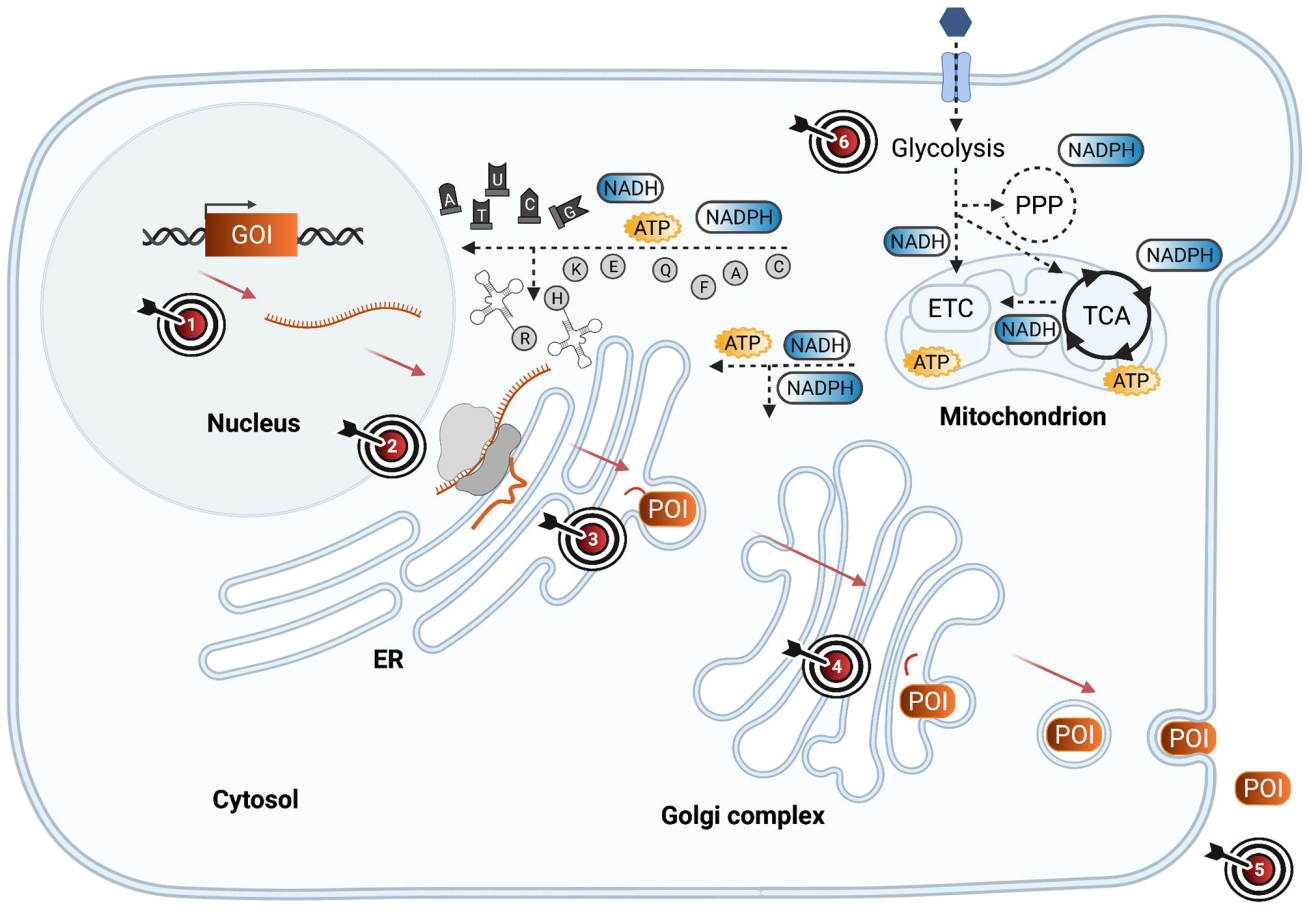
Burden-triggering bottlenecks in yeast cell factories during heterologous protein synthesis and secretion. Schematic representation of yeast cell factory engineered to synthesize and secrete a heterologous protein of interest (POI), encoded by a gene of interest (GOI). The GOI is transcribed into messenger RNA (mRNA), later translated by ribosomes into a peptide that is translocated into endoplasmic reticulum (ER), where it is folded and modified. Via anterograde transport, the POI reaches the golgi complex for further modifications and secretion signal cleavage. Via vesicle exocytosis, the POI is secreted to the extracellular medium. Meanwhile, heterologous protein production is fueled by metabolic pathways such as glycolysis, pentose phosphate pathway (PPP), the tricarboxylic acid cycle (TCA), and the electron transport chain (ETC) which deliver energy (ATP), redox cofactors (NADPH, NADH), nucleotides, and amino acids required for heterologous protein production. Different engineering targets (ETs) that have been highlighted in literature to mitigate the burden that heterologous protein production imposes on the host cell are marked with a target symbol. ET1: Gene dosage, promoter strength, plasmid vs. genome integration. ET2: Codon optimization, co- or post-translational translocation. ET3: Tuning genes involved in unfolded protein response, oxidative stress response, ER-associated protein degradation pathways. ET4: Direct POI for secretion or express intracellularly. ET5: Process engineering by adding certain amino acids to the medium, changing the medium or carbon source. ET6: Redirecting metabolic fluxes by tuning relevant target genes. Synthetic circuit engineering for dynamic regulation may be simultaneously aimed at any combination of ETs 1–4 and/or 6.

Figure 1 illustrates critical steps for the production of heterologous proteins in yeast cell factories and highlights engineering targets for mitigating burden. In the following sections, we will review how burden manifests in production cells from heterologous DNA to secreted protein product.

### The Impacts of Heterologous DNA on Production Strains

Heterologous DNA is maintained in host cells on plasmids or as genome-integrated expression cassettes. Although the energetic cost of replicating extra plasmid DNA is deemed negligible in haploid *S. cerevisiae* (Krogh et al., 2008; Rugbjerg and Sommer, 2019), researchers studying the instability of plasmid copy number in *S. cerevisiae* in both haploid and diploid strains producing heterologous aprotinin found that diploid cells adapted to burden through plasmid loss (Krogh et al., 2008). In contrast, despite an observed decrease in aprotinin production, haploid strains maintained constant plasmid copy number while adapting to extended growth in minimal medium (Krogh et al., 2008). We expect this to be similar in *K. phaffii* but to our knowledge, no research is available in this organism on this topic. However, genomic integration is a more common approach in *K. phaffii*, which we will get back to. Maintaining plasmids, often requires selection marker expression, such as genes encoding for antibiotic resistance or metabolic enzymes, the expression of such drains even more resources from cells and adds to burden (Karim et al., 2013; He et al., 2020; Shen et al., 2021). Therefore, chromosomal integration of heterologous genes without the need for selection markers can help mitigate the fitness cost associated with carrying high-copy number plasmids (Harrison et al., 2012; Vieira Gomes et al., 2018). Alternatively, a weaker promoter driving expression of the selection marker can be utilized, which can help relieve burden as recently reported in *K. phaffii* (Shen et al., 2021). In this case, a 3.3-fold higher plasmid copy number and significantly higher expression levels of insulin precursor were achieved when employing the 300 bp promoter region upstream 2-deoxyglucose-6-phosphate phosphatase gene (P_Dog2p300_), compared to the popular *TEF1* promoter (Shen et al., 2021).

In *S. cerevisiae* production strains, plasmid-based expression and chromosomal integration strategies are both widely employed for heterologous protein production. For *K. phaffii*, chromosomal integration of expression cassettes is preferred since plasmids are much less stable in this species. However, high-copy integration strains can be genetically unstable, likely as a consequence of increased burden from heterologous protein production (Zhu et al., 2009; Yu et al., 2020). In rare instances, loss of gene copies through recombination events in genetically unstable cells can occur, providing a growth advantage over burdened cells with higher heterologous protein expressions levels (Zhu et al., 2009). Thus, chromosomal integrations provide many advantages for heterologous protein expression in both *S. cerevisiae* and *K. phaffii*, but they also present potential challenges if copies of identical sequences or similar genetic elements are used.

The primary biosynthetic cost from high-copy plasmids or chromosomal integrations encoding heterologous genes is protein overexpression rather than the cost of replication (Kim et al., 2001; Harrison et al., 2012; Eguchi et al., 2018; Huang et al., 2018; He et al., 2020; Yu et al., 2020). Consistent with this, a non-linear correlation between protein production and gene dosage is often observed in yeast production strains (Mellitzer et al., 2014; Cámara et al., 2016; Fang et al., 2017; Huang et al., 2018; He et al., 2020). The optimal threshold for gene dosage varies between studies and depends on the protein of interest (Mellitzer et al., 2012). For example, a two-copy strain of *K. phaffii* producing *Rhizopus oryzae* lipase performed better (12.73UA-10^12^/cell) than strains with more than two copies (8.19-12.09UA-10^12^/cell) (Cámara et al., 2016). In another study, a 10-copy strain of *K. phaffii* producing 1.2 g/L *Thermomyces lanuginosus* xylanase A performed better than 6- and 18-copy strains producing ~0.85 and 0.25 g/L, respectively (Mellitzer et al., 2012). In a third study, *K. phaffii* production of the porcine insulin precursor reached a maximum of 0.9 g/L at 6 copies, showing approximately the same production titers for 6, 12, and 18 copies (Chen et al., 2017). These findings indicate that cells possess mechanisms for downregulating heterologous genes when reaching a certain gene dosage plateau, a Goldilocks range of maximum expression, above which burden is triggered (Mellitzer et al., 2014).

Strategies for delivering and maintaining heterologous DNA in production strains, as discussed above, correspond to engineering target 1 in Figure 1. Overall, plasmids or integrated genes in high copy number present potential sources of strain instability. Heterologous DNA maintenance also clearly requires cellular resources from the host. However, the biosynthetic cost of protein production from such foreign DNA has a significantly larger impact on triggering a burden state in the production host. This can be divided into the different steps linked to metabolic pathways, which we will discuss in the following sections.

### Transcription of Heterologous Genes: A Source of Potential Burden

The biosynthetic process of transcription has been reported to be a limiting process for growing *S. cerevisiae* and *K. phaffii* under certain conditions, including in phosphate limiting conditions or when grown in standard medium with increasing gene-copy number (Kafri et al., 2016; Cámara et al., 2017; Farkas et al., 2018). Engineering target 1 in Figure 1 also highlights the potential strategies for controlling optimal heterologous gene expression. It is conceivable that burden could be induced when employing strong promoters due to competition from the heterologous gene promoter for essential transcription factors against native promoters, such as the *K. phaffii* transcription factor Mxr1p responsible for stimulating expression from the *AOX1* promoter (Görgens et al., 2001; Cámara et al., 2017). It is equally possible that high expression levels of the heterologous gene drain other transcriptional resources, such as the transcriptional machinery or ribonucleotides, thereby reducing growth rates and biomass yield (Görgens et al., 2001; Cámara et al., 2017; Rugbjerg and Sommer, 2019). Having said that, eukaryotic genomes are widely transcribed, including many rapidly degraded non-coding RNAs from intergenic and anti-sense regions (Jensen et al., 2013), implying additional transcription from a heterologous gene present in one or more copies should have a negligible impact (cellular protein content is 5–10-fold higher than nucleic acid content). Instead, burden from heterologous genes and their strong transcription is more likely indirect since the mRNAs produced might overload costly downstream processes involved in protein synthesis (Saeki et al., 2020). Nevertheless, increased transcriptional activity of recombinant genes likely contributes, however modestly, to burden (Farkas et al., 2018).

### Protein Synthesis and tRNA Availability Place a Burden on Production Strains

Protein translation is a costly process consuming energy, nutrients, and other resources. Overall, this step is reported to be an important limiting process for protein production in yeast (Kafri et al., 2016; Cámara et al., 2017). Although different resources are critical during translation, the most obvious components for this process include ribosomes and amino acyl tRNAs. Intuitively, high expression levels from heterologous genes leaves fewer ribosomes available to translate native proteins, eventually leading to growth defects (Saeki et al., 2020). However, multiple studies in yeast suggest that the major limiting resources during translation are in fact metabolic products like energy and metabolic precursors, with the occupation of the translational machinery representing a minor source of burden (Heyland et al., 2011b; Kafri et al., 2016). Thus, identifying and enhancing the availability of limiting resources for protein synthesis is expected to reduce burden and enhance overall protein production. This critical step corresponds to engineering target 2 in Figure 1.

The availability of certain tRNAs can also be limited during translation. Aminoacyl-tRNAs formation consumes considerable amounts of cellular energy through hydrolyzation of ATP molecules, while GTP molecules are also subsequently required for amino acid polymerization (Schimmel, 1993). We will discuss causative factors of energy limitations later in this review. The copy number for genes encoding different tRNAs controls to some extent the availability of tRNAs for translation. tRNA gene copy numbers encoding specific anti-codons varies almost 20-fold in *S. cerevisiae* (Chan and Lowe, 2016) and 10-fold in *K. phaffii* (De Schutter et al., 2009). Thus, codon optimizing a heterologous gene is expected to be important to avoid the depletion of specific tRNAs, especially rare ones. Based on differing tRNA gene copy numbers in *S. cerevisiae* and *K. phaffii*, it is however plausible that cognate tRNA availability is a more important consideration for burden mitigation in *S. cerevisiae* production strains. Indeed, tRNA availability and recharging has been suggested to be a non-limiting factor in burdened *K. phaffii* (Mellitzer et al., 2014). Mellitzer et al. applied different codon optimization strategies and observed that certain genes resulted in higher titers independent of the promoter used to drive transcription and carbon source used, conditions under which the aminacyl-tRNA pool was otherwise expected to change and thus have an influence on what optimized genes would result in the highest protein titer or burden the translation machinery. Changes in tRNA concentrations can impact cellular mRNA expression profiles by altering mRNA stability (Presnyak et al., 2015). With this parameter also comes translational speed, which can indirectly contribute to burden since fast translation kinetics can cause incorrect protein folding triggering cellular stress responses (Drummond and Wilke, 2008; Yang et al., 2014). For example, slowing translation through the use of less “optimal” codons can help correct protein folding for some difficult to express heterologous proteins (Mellitzer et al., 2014; Yang et al., 2014). On the other hand, Mellitzer et al. observed a 23-fold difference in expression levels for cellobiohydrolase 2 with different synonymous codon changes in *K. phaffii* (Mellitzer et al., 2014).

Notably, if codon optimality is around the average tRNA adaptation index score [tAI score developed by dos Reis et al. (2004)] of a gene in the specific host organism−0.37 in *S. cerevisiae* (Eguchi et al., 2018); 0.47 in *K. phaffii* (Xu et al., 2021)—the translation elongation rate is often not high enough for an overexpressed protein to reach the burden-limit triggering growth defects. Authors using *S. cerevisiae* as a model organism estimate this to be around 15% of total cellular protein (Eguchi et al., 2018). Similar estimations for *K. phaffii* have not yet been experimentally determined or modeled. Whether tRNA abundance is a factor that should be considered in burdened yeast cell factories and the extent to which codon optimization is a useful strategy to significantly mitigate burden remains unanswered in most cases.

### Amino Acid Supply as a Limiting Factor During Translation

As highlighted above, heterologous gene expression triggers an increased demand for nucleotides, tRNAs, and amino acids, removing resources from the pool of free intracellular building blocks required for mRNA and protein synthesis (Gonzalez et al., 2003; de Ruijter et al., 2018). By comparing *S. cerevisiae* metabolite profiles or flux distribution in production strains to wild type, an increased rate of consumed amino acids has been observed (Gonzalez et al., 2003; de Ruijter et al., 2018). This leaves fewer metabolic precursors for downstream pathways like the tricarboxylic acid cycle (TCA) cycle and pentose phosphate pathway (PPP), leading to decreased flux into energy and reducing power formation. This explains decreased growth rates observed and why supplementing cultivation medium with certain amino acids improves heterologous protein production (Görgens et al., 2005; Heyland et al., 2011b; Van Rensburg et al., 2012; Liu et al., 2016; Huang et al., 2017).

Glutamate and glutamine are important building blocks for nascent polypeptides and amino donors for the biosynthesis of other amino acids (Huang et al., 2017). By supplementing these two amino acids in growth medium, their increased availability helps relieve metabolic limitations by contributing also as additional carbon sources (Heyland et al., 2011b; Nie et al., 2014). Addition of glutamine alone to standard defined medium was reported to cause an increase in β-aminopeptidase 3-2W4 BapA from 0.37 to 0.42 mg/g (Heyland et al., 2011b), whereas in another experiment addition of glutamate caused an increase in β-galactosidase (in this case measured as activity) from 14,570 to 20,460 U/g_DCW_ (Nie et al., 2014). In line with this, RNA-seq revealed that genes involved in *de novo* biosynthesis of glutamate and glutamine were upregulated despite supplementation, as well as amino group transfer genes to help convert them into other amino acids and genes encoding membrane transporters (Huang et al., 2017). Thus, glutamine and glutamate especially might play a central role in adapting to the changing environment, but also amino acid composition of the heterologous protein produced might change the need and limitations in the burdened host cells. Huang and coworkers also found a significant upregulation of YCT1 and ERC1 gene transcription encoding cysteine transporters. In their study they produced a-amylase which amino acid composition has a 9.3-fold higher requirement for cysteine compared to that of the average yeast cell protein (Huang et al., 2017) indicating amino acid composition can affect limitations related to amino acids. On the other hand, the authors of another study, producing insulin precursor from *S. cerevisiae*, found that heterologous protein production had an impact on the cellular free amino acid pool, but that this had no correlation with the relative amino acid abundance in native yeast protein and insulin precursor protein (Kazemi Seresht et al., 2013). Thus, cells appear to adapt to changing demands from heterologous protein production by enhancing both amino acid uptake via membrane transporters as well as *de novo* synthesis.

Glutamate addition changes flux distributions of central carbon metabolism and increases the pool of intermediates in the TCA cycle to improve energy generation in *K. phaffii* (Nie et al., 2014; Liu et al., 2016). Notably, TCA cycle activity appears to react differently in *K. phaffii* and *S. cerevisiae*. For *K. phaffii*, an upper limit of absolute TCA cycle activity to catabolize carbon was reported (Heyland et al., 2011b). Thus, the capacity of the TCA cycle is not always robust enough to catabolize sufficient carbon to meet the increased demands for energy, which will limit growth rates and protein production in *K. phaffi* (Heyland et al., 2011b). On the contrary, the relative TCA cycle flux for *S*. *cerevisiae* correlates with glucose uptake (Blank and Sauer, 2004). Therefore, *S*. *cerevisiae* might not have such an upper limit as has been reported for *K. phaffii*, highlighting a potentially important metabolic difference to consider when engineering more resilient strains of each organism.

In summary, amino acids play an essential role in protein production as both building blocks as well as additional carbon sources for central carbon metabolism. Evidence supports supplementation of certain amino acids in growth medium to alleviate burden in yeast cell factories, as indicated for engineering strategy 5 in Figure 1. Particularly, glutamate and glutamine enhance protein production by relieving metabolic limitation as an added nitrogen and carbon source. However, due to the economic cost of using such supplementations in large-scale bioreactors (Heyland et al., 2011b), this process engineering strategy can be suboptimal at industry-level. Instead, genetic engineering strategies to overcome or bypass such metabolic limitations likely offer more cost-effective alternatives to mitigate burden at this stage in the protein production process.

### NADPH Demands During Translation Contribute to Burden

High demand for amino acids can also contribute to establishing a burden state in production cells due to limitations in the redox cofactor nicotinamide adenine dinucleotide phosphate (NADPH). NADPH is required in many anabolic processes, including *de novo* synthesis of amino acids, and demand for this redox cofactor predictably rises in production strains (Zahrl et al., 2019).

NADPH can be generated through two main pathways: the oxidative phase of PPP or the acetate formation pathway (Grabowska and Chelstowska, 2003; Kwolek-Mirek et al., 2019). Primary generation of NADPH via PPP was reported for burdened *K. phaffii* production strains where acetate did not accumulate despite increased demands for NADPH (Nie et al., 2014). Therefore, upregulating genes in the oxidative phase of PPP might be expected to help cells meet the growing demand for NADPH. Such approaches are highlighted as engineering strategy 6 in Figure 1 and have been confirmed in *K. phaffii* strains producing human superoxide dismutase, whereby enhancing the first two steps in the oxidative phase of PPP proved to increase production by 3.8-fold (Nocon et al., 2016). However, it did not alleviate the observed decreases in specific growth rate and biomass yield. This potential solution might not work in burdened *S. cerevisiae* strains since RNA-seq and proteomic analyses show that genes involved in PPP are not upregulated, but rather downregulated (Huang et al., 2017; Wright et al., 2020). Instead, increased acetate formation has been reported from an insulin-producing *S. cerevisiae* strain, indicating preferential NADPH production via the acetate formation pathway to meet increased demand resulting from augmented amino acids needs (Wright et al., 2020). Others however argue that *S. cerevisiae* production strains meet NAPDH demands by simply reducing biomass yield in general (Huang et al., 2017).

In contrast to *S. cerevisiae*, researchers studying protein production in *K. phaffii* observed an increase in biomass yield (from 0.39 to 0.49 g_CDW_${\text{g}}_{\text{glucose}}^{-1}$), and a decrease in growth rate (from 0.3 to 0.18h^−1^) and glucose uptake rate (from 4.2 to 2.2 mmol g^−1^h^−1^) for BapA production strains compared to the reference strain (Heyland et al., 2011b). The PPP flux correlated with biomass yield, whereas correlations between PPP and protein production were low. This indicates a higher demand for NADPH due to an increase in biomass synthesis rather than protein production. In this context, it is also important to mention that flux through PPP also depends on substrates used for cultivation. In general, methylotrophic yeasts like *K. phaffii* have retained high PPP activity during evolution (Riley et al., 2016). The assimilation of methanol requires a higher carbon flux through PPP (Jordà et al., 2012) since the ketose sugar PPP intermediate, xylulose 5-phosphate, is needed to drive this reaction. Thus, increased PPP flux does not only reflect higher NADPH demand in cases where methanol is used during cultivation (Nie et al., 2014). Meanwhile, *S. cerevisiae* is unable to grow on methanol, using simple sugars such as glucose as carbon sources.

In summary, these two organisms rely on different pathways for NADPH production in burdened production strains: the acetate formation pathway is favored in *S. cerevisiae*, while the oxidative phase of PPP in *K. phaffii*.

### General Challenges With Redox Imbalance in Burdened Production Strains

Another important redox cofactor in cells is NAD+/NADH. During oxidative phosphorylation in mitochondria, NADH is required for ATP energy formation through the respiratory chain reaction. ATP is needed for almost all growth-related activities, cellular maintenance, and heterologous protein production. Hence, the burden from heterologous protein production increases the demand for carbon allocated to the TCA cycle and oxidative phosphorylation for ATP production (Heyland et al., 2010). For this reason, ATP is likely to decrease in high producing strains as a response to burdensome protein production, compensated by slower growth and carbon uptake (Tyo et al., 2012; Nie et al., 2014). This further highlights the central role of carbon metabolism on burden.

High NADH demand in production strains requires greater redox balance. Under aerobic conditions, *S. cerevisiae* and *K. phaffii* can use glycerol as an electron acceptor to oxidize NADH. This was used as an explanation for glycerol production in *K. phaffii* to help maintain redox balance and avoid cytosolic NAD+ depletion (Nie et al., 2014). In the same study, the authors also found that higher producing strains reduced by-product formation, such as glycerol production, and concluded this is a strategy for the cell to compensate for a higher demand of redox-cofactors and energy (Nie et al., 2014). In line with this, an increase in glycerol production can also lead to lower biomass yield as carbon is removed from central carbon metabolism, contributing to burden (Krogh et al., 2008). Of note for *K. phaffii*, problems associated with NADH limitations will likely be worse in glucose cultivation as opposed to growth on methanol in co-fed cultures because of the energetic regulation of enzymes involved in methanol oxidation and assimilation pathway (Jordà et al., 2012). While redox requirements for translation are higher in production strains, post-translational processes also require redox factors and other resources. The impact on burden from post-translational processes is discussed next.

### Burdensome Protein Folding, Export, and Secretion Triggers Cellular Stress Responses

Secretion of post-translationally modified proteins by yeast cell factories is advantageous as it facilitates simple downstream purification and processing steps (Nielsen, 2013). The downside is that it increases demand for heterologous protein translocation into the endoplasmic reticulum (ER), folding in the ER, and export via the Golgi apparatus. Together this can introduce bottlenecks that place additive burden on yeast cell factories and trigger cellular stress responses (Jordà et al., 2012; Tyo et al., 2012; Huang et al., 2017; de Ruijter et al., 2018). Indeed, the impact of post-translational processes on the burden state is thought to be a major limitation in yeast, especially considering relatively lower expression levels for these organisms compared to bacteria (Mattanovich et al., 2004).

Proteins are modified, folded, and transported in the ER. Yeast cells must compensate for the burden conferred by the energy-consuming secretory pathway. This is achieved by allocating more resources to cellular maintenance for energy production and away from growth (Jordà et al., 2012; Chen et al., 2020). Even when directing expressed proteins to mitochondria, in contrast to cellular secretion, resources for transportation are a limiting factor causing plasmid expression repression and growth defects (Kintaka et al., 2016; Eguchi et al., 2018). Similarly, anchoring heterologous β-glucosidase to the yeast cell membrane induces burden (Ding et al., 2018). Thus, in order to fully understand burden in yeast, we must better elucidate the burdensome steps during protein transportation and secretion.

#### Translocation Into the ER

Once a heterologous protein has been effectively translated in yeast, the nascent peptide is translocated into the ER. The timing of nascent peptide translocation, e.g., co- or post-translational, can affect burden on yeast cells (Tang et al., 2015; Barrero et al., 2021) and is directed by the pre-sequence of the leader (Hou et al., 2012). For example, the *K. phaffii* pre-alpha-mating factor (MF), which is commonly fused to heterologous proteins to drive their post-translational translocation into the ER, augmented burden during *Rhizopus oryzae* lipase (ROL) expression. This was observed as a reduction in growth rate, biomass yield, and final protein titer (Barrero et al., 2021). Notably, burden was less pronounced when using an alternative translocation signal called pre-Ost which stimulates co-translational translocation of the nascent peptide into ER. In fact, compared to ROL fused to pre-alpha-MF signal, the final ROL titer was improved from 192.4 to 291.9 U/ml (Barrero et al., 2021). Similarly, co-translational translocation enhanced secretion from *S. cerevisiae* production stains expressing β-glucosidase, endoglucanase, and α-amylase (Tang et al., 2015). In some cases, native leader sequences will be advantageous to use instead of yeast-specific leaders as for example reported for human serum albumin (Sleep et al., 1990). However, predicting which leader sequence most efficiently facilitates translocation into ER and further secretion is difficult (Hou et al., 2012). The use of non-efficient leader sequences for directing a heterologous protein through the secretory pathway can contribute to burden. Secretion via co-translational translocation of heterologous proteins into the ER, in contrast to post-translational translocation, was reported to alleviate some of the burden introduced at this step and can be a strategy to mitigate burden. Such strategy is highlighted as engineering target 2 in Figure 1.

#### Protein Folding in the ER

Major resource consuming steps in the secretory pathway include protein folding and disulfide bond formation, a common stabilizing feature in many secreted proteins. Protein chaperones consume significant amounts of ATP to facilitate protein folding and thereby prevent their aggregation and/or degradation (Umebayashi et al., 1997; Walter and Buchner, 2002; Hartl et al., 2011). Despite added energy demands, co-expressing certain chaperones in yeast production strains has been shown to enhance heterologous protein by helping to overcome burden at this crucial folding step (Zhang et al., 2006; Gu et al., 2015; Yu et al., 2017). Addition of affinity tags, usually added for detection and purification, can also help facilitate folding. These fusion partners might however induce burden at different levels, such as widely used His-6 tag is proposed to induce low burden and glutathione S-transferase tag to induce high burden (Waugh, 2005). Therefore, it is advised to consider this when adding affinity tags.

The formation of disulfide bonds in the folding process is redox-driven, which can lead to severe oxidative stress in production cells if imbalanced (Margittai and Sitia, 2011). De Ruijter et al. recently reported that production of heterologous antibody fragments in *S. cerevisiae*, which require disulfide bonds, clearly burdened ER redox balance and increased flux of glutathione metabolism (de Ruijter et al., 2018). This is because protein folding becomes slower than disulfide bond formation as the protein folding machinery becomes overloaded and this will consume glutathione for disulfide bond breaking, producing reactive oxygen species (ROS) that induce oxidative stress (Tyo et al., 2012). This has also been proposed as an explanation for proteome changes over time in *S. cerevisiae* cells burdened by insulin production, which also requires disulfide bond formation (Wright et al., 2020). In line with this, overexpressing glutathione peroxidase 1, the enzyme responsible for detoxifying ROS from reduced glutathione, was observed to improve disulfide bond-containing AppA phytase production by 1.3-fold (from ~350 mU/ml) in *K. phaffii* (Navone et al., 2021). Alternatively, in another study burden was mitigated by over-expressing HAP1 gene encoding a transcription factor activating oxidative stress response genes and helped to increase growth rate and respiration. This alpha-amylase producing *S. cerevisiae* strain performed better (45 U${\text{g}}_{\text{DCW}}^{-1}$h^−1^) than the reference strain (34 U${\text{g}}_{\text{DCW}}^{-1}$h^−1^) in a chemostat with dilution rate near the maximum of the reference strain but still far from maximum for the optimized strain (Martínez et al., 2016). Such approaches correspond to engineering target 3 in Figure 1.

Engineering strategies focused on co-expressing protein chaperones, peroxidases or transcription factors to resolve secretory pathway bottlenecks might seem counter-intuitive, since expressing additional proteins might be expected to have an additive effect on burden by competing for some of the same translational and post-translational resources as the heterologous protein (Liu et al., 2014; Huang et al., 2018; Yu et al., 2020). This can explain why such strategies do not always work or sometimes only modestly enhance expression (Yu et al., 2017). Tightly regulating and fine-tuning the overall amount and timing of expression for such proteins is expected to have better results (Navone et al., 2021) than utilizing high copy number plasmids for over-expression (Huang et al., 2018).

The cofactor NADPH is also used as a reducing agent of glutathione disulfide to regenerate reduced glutathione (Ayer et al., 2014). The need for NADPH was suggested to cause similar levels of flux through the oxidative PPP branch in lipase-producing *K. phaffii* compared to the reference strain, despite lower biomass yield in the production strains. The high PPP flux ensures adequate levels of the glutathione electron donor for protein folding (Jordà et al., 2012). Moreover, for an *S. cerevisiae* strain producing a human insulin precursor, burden-induced lower PPP flux was considered to have a negative impact on folding in ER, because another enzyme involved in disulfide bond formation, termed protein disulfide isomerase (PDI1), also requires NADPH (Kazemi Seresht et al., 2013). Therefore, NADPH limitations become more problematic in production strains engineered to secrete proteins, as opposed to intracellularly expressed proteins, due to the additional demand for NADPH during later export processes after protein folding in ER (Liu et al., 2016). While tuning the oxidative phase of PPP in burdened *K. phaffii* enhances heterologous protein production (Nocon et al., 2016), it is currently unclear whether this PPP engineering strategy would improve production in *S. cerevisiae*.

#### Quality Control Systems in the ER

Burden associated with increased oxidative stress response and the accumulation of unfolded protein in ER can trigger the activation of the unfolded protein response (UPR). UPR is a quality control system that helps re-establish cellular proteome homeostasis and is therefore activated as a response to burden in the ER (Huang et al., 2017; Chen et al., 2020). While chaperones that act on misfolded proteins may buffer against burden (Farkas et al., 2018), UPR acts as a defense system against burden. However, in some instances UPR activation might contribute additional burden on cells (Lamour et al., 2019), due to the upregulation in many UPR genes (de Ruijter et al., 2018). In contrast to this view, a recent study in *K. phaffii* found that proteins involved in UPR decreased when switching from glycerol batch pre-cultivation to methanol fed-batch (Vanz et al., 2014). Glycerol cultivation is associated with high UPR induction, and although cultivation in methanol does not induce the same level of UPR activation, the authors suggest that heterologous protein production may benefit from initially high UPR activity. In line with this, the basal UPR level in *K. phaffii* appears to be higher compared to other yeasts (Vanz et al., 2014). Therefore, enhanced UPR activity or the co-expression of UPR factors might mitigate burden upon the strong induction of heterologous protein synthesis following the switch to methanol as a carbon source.

The main regulator of UPR in yeast is the transcription factor Homologous to Atf/Creb1 (HAC1). Prolonged overexpressing of this transcription factor improved production in burdened *K. phaffii* strains, increasing heterologous Xylanase from ~140 to 200 U/ml and glucose oxidase from ~55 to 140 U/ml (Lin et al., 2013; Yu et al., 2020). However, it also reduced cell growth. The negative correlation between protein secretion capacity and cellular growth was ascribed to stress but meets our definition of burden. And despite efforts to reduce burden with this strategy, the co-expression of a heterologous protein and the UPR regulator was able to enhance expression but contributed its own burden (Yu et al., 2020). This might be explained by the fact that increased ROS levels were associated with HAC1 overexpression, suggesting that prolonged UPR activation triggered oxidative stress (Yu et al., 2020). Therefore, while this strategy successfully enhanced heterologous protein expression, a new source of burden is elicited.

In addition to UPR, the ER-associated-degradation (ERAD) pathway is another important quality control system in yeast. ERAD targets misfolded proteins for cytosolic degradation and ensures that ER homeostasis is maintained (Hwang and Qi, 2018). Co-expressing ERAD-related genes has been explored as a strategy to mitigate burden in yeast. For example, co-expressing the ERAD ubiquitin ligase called HMG-coA Reductase Degradation (HRD1) together with a heterologous glucose oxidase helped limit burden in *K. phaffii* (Gu et al., 2015). While co-expressing single genes such as HRD1 or HAC1 alleviates some of the cellular stress induced by protein folding burden, yeast can adapt to prolonged ER stress induced by folding burden through chromosomal duplication (Beaupere et al., 2018; Beaupere and Labunskyy, 2019). Such genomic instability might present additional problems for production strains, indicating that more tightly controlled regulatory circuits that selectively turn specific genes in the UPR and ERAD pathways on or off might be more desirable than constitutively over-expressing them.

To summarize, cellular stress is tightly coupled to burden. As heterologous proteins are directed to the secretory pathway, burden is induced by overload and subsequent disruptions in protein folding and redox balance in the ER which trigger the onset of different cellular stress responses. Engineering target 3 in Figure 1 highlights this important step. Evidence supports increased heterologous protein titers when co-expressing one or a few specific stress-response proteins, indicating that stress-response pathways provide attractive targets for engineering. However, more robust, and tightly regulated circuits can potentially be more efficient to mitigate burden accumulation and allow cells to maintain a healthier homeostasis.

## Future Profiling and Engineering Strategies

Several process and genetic engineering strategies have been proposed to improve production from burdened host cells (Deparis et al., 2017). We summarize these in Figure 1 and Table 2. These strategies encompass: enhancing the availability of certain co-factors; improving protein folding and/or folding capacity in the ER; optimizing gene copy number and codon usage; engineering improved gene promoters; and finally, optimizing cultivation conditions to support increased resource demand. Moving forward, engineering metabolic networks represents a promising approach to improve heterologous protein titers by helping alleviate redox cofactor and energy limitations in burdened cells, such as leading flux toward necessary precursors (Mattanovich et al., 2014; Nie et al., 2014).

**Table 2:** Strategies to improve production from burdened yeast cell factories.

**Engineering strategy**	**Strategy category**	**Host**	**Heterologous protein**	**Production**	**Optimized production**	**Fold improvement**	**Reference**
Changing medium from BMGY to rich defined medium	P	*Kp*	Human growth hormone	~0.0221 *g*· *L*^−1^	~ 0.201 *g*· *L*^−1^	9	Matthews et al., 2018
Copy number optimized from 3 to 4 copies, codon optimized, and stronger promoter	G	*Kp*	*Trichoderma reesei* Cellobiohydrolase 2	~2.9 *g*· *L*^−1^	~15.7 *g*· *L*^−1^	5.4	Mellitzer et al., 2014
Co-factor upregulation: Overexpression of PPP-related ZWF1 and SOL3	G	*Kp*	Human superoxide dismutase	24.4 *mg*·*g*^−1^*	73.2−97.6 *mg*·*g*^−1^	3**–**4	Nocon et al., 2016
Amino acids supplementation to chemically defined medium	P	*Sc*	β-glucosidase	0.036 *g*· *L*^−1^	0.1 *g*· *L*^−1^	2.8	Van Rensburg et al., 2012
Overexpression of ERAD-related ubiquitin ligase Hrd1	G	*Kp*	Glucose oxidase	6.18 *g*·*L*^−1^	11.08 *g*·*L*^−1^	1.8	Gu et al., 2015
Sorbitol/methanol co-feeding instead of methanol alone	P	*Kp*	Porcine insulin precursor	~0.6 *g*·*L*^−1^	~0.9 *g*·*L*^−1^	1.5	Chen et al., 2017
Exchanging pre-alpha-MF ER translocation signal sequence with pre-OST1	G	*Kp*	*Rhizopus oryzae* lipase	192.4 *U*·*mL*^−1^	291.9 *U*·*mL*^−1^	1.5	Barrero et al., 2021
Overexpression of TF Fhl1 involved in ribosome biosynthesis processing	G	*Kp*	Pectinase (intracellularly)	180 *U*·*mL*^−1^	250 *U*·*mL*^−1^	1.4	Zheng et al., 2019
Constitutive Hac1 expression for UPR upregulation	G	*Kp*	Xylanase A	~140 *U*·*mL*^−1^	~200 *U*·*mL*^−1^	1.4	Lin et al., 2013
Addition of glutamate to the medium	P	*Kp*	β-galactosidase	0.128 *mg*·*g*^−1^	0.165 *mg*·*g*^−1^	1.3	Liu et al., 2016
Minimal medium supplemented with all 20 proteinogenic amino acids	P	*Kp*	β-aminopeptidase 3-2W4	37 *mg*·*g*^−1^	47 *mg*·*g*^−1^	1.3	Heyland et al., 2011b
Driving transcription of target genes endogenously with strong promoters instead of plasmid-borne expression	G	*Sc*	a-amylase	17.5 *mg*·*g*^−1^	23 *mg*·*g*^−1^	1.3	Huang et al., 2018
Over-expression of Hap1 increasing oxidative stress response	G	*Sc*	a-amylase	$34\;U\cdot g_{DCW}^{-1}\cdot h^{-1}$	$45\;U\cdot g_{DCW}^{-1}\cdot \;h^{-1}$	1.3	Martínez et al., 2016

* *mg/g indicates mg protein of interest per g total cellular protein*.

*BMGY, Buffered Glycerol Complex Medium; ER, Endoplasmic Reticulum; ERAD, ER-Associated Protein Degradation; Fhl1, Fork Head-Like; G, genetic Engineering; Hac1, Homologous to Atf/Creb1; Hap1, Heme Activator Protein; Hrd1, HMG-coA Reductase Degradation; Kp, Komagataella phaffii; MF, Mating Factor; OST1, OligoSaccharylTransferase; P, Process Engineering; PPP, Pentose Phosphate Pathway; Sc, Saccharomyces cerevisiae; SOL3, Suppressor Of Los1-1; TF, Transcription Factor; UPR, Unfolded Protein Response; ZWF1, ZWischenFerment*.

Systems biology offers a promising interdisciplinary approach to discover new targets for metabolic engineering (Hou et al., 2012). Apart from analyzing systems level data, it can also be integrated into mechanistic models. Combining *in silico* modeling with -omics techniques and bioinformatic analyses allows for the identification of factors that are critical but might not necessarily stand out when the data is first observed. Thus, modeling represents a useful tool for quantifying the bioproduction limitations of living cells (Mattanovich et al., 2014; Volkova et al., 2020; Patra et al., 2021). Constraint-based stoichiometric models, in particular, can be used to calculate the metabolic differences between strains and allow for quantification of burden on metabolism (Gonzalez et al., 2003). For example, carbon flux distribution in central carbon metabolic pathways can be traced by applying C^13^-metabolic flux analysis and additional *in silico* data into stoichiometric models (Jordà et al., 2012; Nie et al., 2014). To broaden the applicability, burden should be addressed and incorporated into such models like large genome-scale models (Saitua et al., 2017).

Indeed, diverse systems-based efforts have been introduced to alleviate burden in production hosts. Notably, such efforts have inspired the construction of dynamic control systems that respond to changing metabolic and energy demands. These have proven highly effective in *E. coli* production strains and show strengths over the more classic genetic engineering approach of simply deleting or over-/under-expressing candidate genes. As heterologous protein production requires resources from shared pools of metabolites with native cellular processes, static overexpression of specific genes leads to a less robust systems since it does not help minimize the changing levels of burden on production host during cultivation (Boo et al., 2019). Instead, dynamic control can stabilize heterologous protein production over time. For example, Ceroni et al. engineered a burden-driven feedback system in *E. coli* using CRISPR technology which dynamically regulated heterologous protein production in response to burden (Ceroni et al., 2018). In this system, transcription of a single guide RNA (sgRNA) is controlled by a burden-responsive promoter identified by RNA-seq analysis. In response to burden, the sgRNA is transcribed and directs a catalytically-dead Cas9 protein (dCas9) to the heterologous gene promoter, suppressing its expression until burden is alleviated (Ceroni et al., 2018). By temporarily reducing heterologous protein production, burdened *E. coli* cells have time to recover and achieve greater production levels over the total course of the cultivation. This study highlights the potential for engineering similar burden-driven negative feedback systems for heterologous protein production in yeast.

Similar and other kinds of regulatory feedback circuits have already proven useful to mitigate or circumvent burden in *S. cerevisiae* strains engineered to produce valuable compounds such as bioethanol or vanillin-β-glucoside (D'Ambrosio et al., 2020; Qin et al., 2020). For bioethanol production in *S. cerevisiae*, a more robust strain was achieved by placing target genes in the ethanol pathway under control of stress-regulated promoters to ensure dynamic feedback regulation (Qin et al., 2020). Alternatively, a stable *S. cerevisiae* cell factory was developed to produce vanillin-β-glucoside by coupling pathway intermediate production to biosensor-controlled expression of an essential gene (D'Ambrosio et al., 2020). The absence of the biosensor-coupled burden pathway intermediate renders host cells inviable. In other words, the cells become addicted to production. Although these two examples highlight alternative engineering strategies to produce valuable chemical compounds in yeast, they also involve tightly regulated changes in protein expression (e.g., enzymes required to synthesize valuable metabolites) in response to burden imposed by production.

The above mentioned strategies represent novel and promising approaches which could be applied for burden-driven regulation of heterologous proteins expression. It might also be useful to place secondary “helper genes” under the control of such dynamic regulation, including genes encoding some combination of factors involved in restoring redox balance, controlling UPR and/or ERAD pathways, or ensuring adequate protein folding. Engineering dynamic systems that tailor heterologous protein expression to ever-changing host cell needs over the course of the cultivation represents a novel approach that aims at multiple engineering targets highlighted in Figure 1 simultaneously. Such an approach is anticipated to generate burden-resistant yeast cell factories to boost biopharmaceutical and industrial enzyme production ensuring more stable production over time.

## Discussion

Burden derived from resource competition is often encountered in bioproduction processes triggering a phenotype with compromised production during long-term cultivations. Therefore, engineering production strains that are either more tolerant to burden or able to adjust heterologous protein expression to burden is paramount, and efforts have already been made to engineer such yeast strains as listed in Table 2. To do so requires a better understanding of the different molecular sources of this phenomenon. A reduction in heterologous protein titer, as a consequence of burden, might happen for different reasons. For example, burden itself can impose selective pressure on cells evolving toward lower levels of production through genetic or non-genetic variation (Rugbjerg and Olsson, 2020). This was recently observed in an isogenic population of insulin-producing *S. cerevisiae* cell factories, where burdensome production and cultivation caused non-genetic heterogeneity in the population to favor the growth of low-producing cells in the population (Wright et al., 2021).

Commonly, the impact of burden is measured as a reduction in physiological parameters like specific growth rate, biomass yield, and respiratory capacity (Kazemi Seresht et al., 2013; Liu et al., 2014; de Ruijter et al., 2018). It is worth mentioning that burden might manifest in different ways depending on scale and mode of fermentation. Experimental setup and fermentation equipment will undoubtedly have an impact on these physiological parameters. While reduced protein titers can be a measure of burden during fermentation more broadly, measures like growth rate and biomass are more relevant in batch-type fermentations (Heyland et al., 2011b; Van Rensburg et al., 2012), often representing a screening scenario for strain development. Transferability of such results to industrially relevant cultivation modes is however difficult. It is questionable whether optimized strains from screening will perform well in fed-batch or continuous fermentation environments (Looser et al., 2014). Proxies for cell maintenance, such as amount of energy that the host cell uses to readjust to protein production, is also relevant to assess burden in controlled and batch fermentations. Further, it will be more relevant to use measures like respiratory quotient, that cannot be accounted for during biomass yield, as a measure for burden in, for example, chemostat cultivation mode (Jordà et al., 2012). To do so, underlying biological processes, such as metabolic fluxes and transcription factor activity, are relevant to quantify.

Sources of host cell burden can be more specifically identified from high-throughput, omics-based approaches since they provide a systems-level overview of the burden response. With tools that profile the metabolome, transcriptome, proteome, and/or fluxome of any specific production strain over time, these complex networks can be dissected to understand the burdened phenotype at a molecular level, identify causal factors responsible for pathway bottleneck, and allow rational engineering strategies to alleviate burden and enhance heterologous protein production (Heyland et al., 2011b; Jordà et al., 2012; Liu et al., 2016; Huang et al., 2017; Wright et al., 2020). While the resource drain originating from all processes during the biosynthetic production of a heterologous protein (transcription, translation, folding, and secretion) can have an impact on overall burden, the main culprit, as we have seen, is often tied back to constraints in central carbon metabolism. This central metabolic network is highly affected by the increased need for energy, redox-cofactors, and free metabolic precursors required for the costly production of an additional non-essential protein (Mattanovich et al., 2014). Therefore, cellular metabolism appears key to understanding the underlying mechanisms for burden state induction in yeast, and therefore suitably defined as metabolic burden.

In line with this, the gene regulatory network of underlying metabolic pathways might play an important role in inducing burden and could be an interesting direction for future studies. As reviewed here, cellular stress responses are intertwined with the burden state. Likely, these stress responses and other pathways have additional functions such as facilitating crosstalk with the metabolic pathways affected by burden (Kalender and Çalik, 2020). For example, UPR is reported to behave as a regulatory network activating cytosolic pathways. In a burdened insulin-producing strain of *S. cerevisiae*, activation of amino acid biosynthesis was suggested to be elicited following the up-regulation of transcription factor Gcn4, which is induced by UPR (Kazemi Seresht et al., 2013). It is expected that Gcn4 has the same properties in *K. phaffii* (Gu et al., 2015). Therefore, elucidating such system-wide interactions and regulatory networks will be critical to fully understand the cellular response to burden.

There are still gaps in our understanding of the concept of burden in yeast cell factories. By emphasizing this topic, we hope to draw the attention of future research to elucidate the molecular basis and underlying mechanisms of burden in yeast to mitigate this common production limitation. Despite these challenges, recent advances in bioengineering are already paving the way for improved heterologous protein titers from yeast cell factories by developing strategies that alleviate burden, such as dynamic control system engineering. We expect such approaches to be adopted by the biotech industry and significantly enhance heterologous protein production in the years to come.

## Author Contributions

CW and MJ conceived the topic reviewed in this manuscript. CW, MJ, RA, and LK contributed content and ideas for the manuscript structure. LK wrote the manuscript. CW, MJ, and RA contributed with corrections and revised drafts at all stages. All authors approved the submitted version.

## Conflict of Interest

The authors declare that the research was conducted in the absence of any commercial or financial relationships that could be construed as a potential conflict of interest.

## Publisher's Note

All claims expressed in this article are solely those of the authors and do not necessarily represent those of their affiliated organizations, or those of the publisher, the editors and the reviewers. Any product that may be evaluated in this article, or claim that may be made by its manufacturer, is not guaranteed or endorsed by the publisher.
